# Characterization of Bacterial Communities from the Surface and Adjacent Bottom Layers of Water in the Billings Reservoir

**DOI:** 10.3390/life12081280

**Published:** 2022-08-22

**Authors:** Marta Angela Marcondes, Andrezza Nascimento, Rodrigo Pessôa, Jefferson Russo Victor, Alberto José da Silva Duarte, Patricia Bianca Clissa, Sabri Saeed Sanabani

**Affiliations:** 1 Post-Graduation Program in Translational Medicine, Department of Medicine, Federal University of São Paulo, São Paulo 04039-002, Brazil; 2 Laboratory of Medical Investigation LIM-56, Division of Clinical Dermatology, Medical School, University of São Paulo, São Paulo 17012-901, Brazil; 3 Immunopathology Laboratory, Butantan Institute, São Paulo 05503-900, Brazil; 4 Laboratory of Medical Investigation LIM-03, Hospital das Clínicas (HCFMU), School of Medicine, University of São Paulo, São Paulo 17012-901, Brazil

**Keywords:** bacterial composition, bacterial diversity, 16S rRNA gene, Illumina MiSeq, billings reservoir

## Abstract

Here, we describe the bacterial diversity and physicochemical properties in freshwater samples from the surface and bottom layers of the Billings Reservoir, the largest open-air storage ecosystem in the São Paulo (Brazil) metropolitan area. Forty-four samples (22 from the surface and 22 from the bottom layers) were characterized based on 16S rRNA gene analysis using Illumina MiSeq. Taxonomical composition revealed an abundance of the *Cyanobacteria* phylum, followed by *Proteobacteria*, which were grouped into 1903 and 2689 different genera in the surface and the deep-water layers, respectively. *Chroobacteria*, *Actinobacteria*, *Betaproteobacteria*, and *Alphaproteobacteria* were the most dominant classes. The Shannon diversity index was in the range of 2.3–5.39 and 4.04–6.86 in the surface and bottom layers, respectively. *Flavobacterium* was the most predominant pathogenic genus. Temperature and phosphorus concentrations were among the most influential factors in shaping the microbial communities of both layers. Predictive functional analysis suggests that the reservoir is enriched in motility genes involved in flagellar assembly. The overall results provide new information on the diversity composition, ecological function, and health risks of the bacterial community detected in the Billings freshwater reservoir. The broad bacterial diversity indicates that the bacterioplankton communities in the reservoir were involved in multiple essential environmental processes.

## 1. Introduction

Access to safe, clean, and affordable water is one of the most basic humanitarian goals and is a major global challenge for the 21st century [1,2]. Both pollution from the outside and pollution from inside the reservoir worsen the water quality [3]. Exogenous contamination comes from sources such as industrial waste; sewage runoff; solid waste; meteoric water; and surface runoff from cities, farms, and forests [4,5,6]. Endogenous pollution comes from sediments, phytoplankton, aquatic plants, and aquaculture. Organic matter and biological and chemical oxygen demand (BOD and COD) in reservoirs can be either allochthonous or autochthonous. While the autochthonous organic matter is produced by phytoplankton and hydrophytes, allochthonous organic matter is introduced into aquatic systems by rainfall runoff [7]. BOD and COD levels indicate reservoir contamination, and rising nutrient levels cause reservoir BOD and COD levels to rise. Reservoirs are critical artificial aquatic bodies for many drinking water supply systems because they can maintain an equilibrium of storing or releasing water and play a central role in biogeochemical cycling, energy flows, and the recycling of nutrients. The functioning of these cycles is mainly maintained by the inhabiting microorganisms. Despite their potential importance, the structure and functioning of these ecosystems’ microbial communities have received less attention than other freshwater bodies, such as natural lakes and rivers [8].

Several studies have analyzed the structure and ecology of bacterial communities and have revealed the unexpected diversity of functional microbes in freshwater ecosystems [9,10,11,12,13,14,15,16]. For instance, a recent study by Shen et al. [17] has revealed the relationships between trophic status and planktonic microbial community structure and metabolic potential in freshwater lakes on the Yun-Gui Plateau, China. Wu K. et al. [18] showed that water depth was the most critical factor affecting the relative abundance of 11 dominant bacterial phyla, including *Proteobacteria* as the most dominant phylum in this lake, followed by *Bacteroidetes*, *Chloroflexi*, and *Nitrospirae* in the sediments of Lake Lugu in Southwest China. An earlier study by De Wever et al. [19] undertook a denaturing gradient gel electrophoresis analysis of PCR-amplified 16S RNA fragments approach to reveal the presence of pronounced vertical and latitudinal gradients in the bacterial community composition in Lake Tanganyika in East Africa. *Actinobacteria* and *Gammaproteobacteria* were found in the epilimnion, whereas *Nitrospirae*, green nonsulfur bacteria, *Gammaproteobacteria*, *Actinobacteria*, *Deltaproteobacteria*, and *Firmicutes* were found in the hypolimnion. The genetic diversity and activity of planktonic bacteria in freshwater systems are strongly shaped by the surrounding environmental conditions. Several studies have demonstrated that bacterial community structures depend on physical conditions and the availability of resources [20,21]. Moreover, it has been demonstrated that the extensive partitioning of water masses in such ecosystems may lead to progressively increased differences in both bacterial diversity [22] and water chemistry [23].

The Billings Reservoir in the Brazilian state of São Paulo is the largest freshwater storage aquatic body in the São Paulo Metropolitan Region, covering 127 km^2^. It has a total volume of 1228.7 × 106 m^3^ and a maximum depth of 18 m [24,25]. The reservoir is used for a variety of purposes, including hydropower generation, water supply for 4.5 million people and industries, irrigation, fisheries, and flood control. The basin has a 20-km-long narrow central body that is connected and fed by eight branches, namely the Rio Grande, Rio Pequeno, Capivari, Pedra Branca, Taquacetuba, Bororé, Cocaia, and Alvarenga rivers [26,27,28]. Since the 1940s, the reservoir properties have changed significantly. At that time, part of the polluted water from the Tiete River (São Paulo city) was allowed to flow into the reservoir, believing that it would raise the water level to generate electric power [29]. This process, along with uncontrolled urban growth, significantly contributed to considerable anthropogenic eutrophication and an algal bloom in the reservoir [30].

There are very few studies on the bacterial community structures and compositions of the surface water in Brazil [31,32]. Thus, the goals of this study were to (i) explore the bacterial communities in surface and bottom layer water along the Billings Reservoir using 16S rRNA gene-based Illumina MiSeq sequencing, (ii) evaluate the presence of potential pathogens in these water samples, and (iii) explore the predicted functional biomarkers of the obtained bacterial communities in the basin to determine their role in the ecosystem.

## 2. Material and Methods

### 2.1. Study Sites and Sample Collection

**Figure 1:**
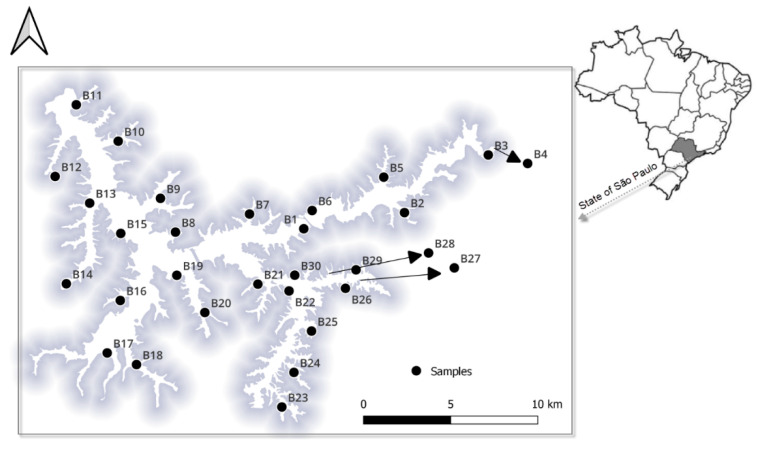
Map showing sampling site locations in the Billings Reservoir in São Paulo. The map was generated using QGIS 3.22.0 (Redlands, CA, USA).

The study area covered the entire 127 km^2^ of the Billings Reservoir, which is located west of the city of Sao Paulo at 23°47′ S, 46°40′ W, at an altitude of 746 m a.s.l. Surface and bottom layer water samples (250 mL each) were collected in July 2019 from 30 locations (approximately 17 km apart; Figure 1) in triplicate using a Van Dorn sampler as previously described [5]. Surface samples were collected from the side of a boat just below the water surface (10 cm) and the bottom water was collected at approximately 0.1 m above the sediment to avoid collecting soil material. Depending on the locations of the collected samples, the water depth of the study area from the reservoir ranged from 1.16 m to 13.6 m. A total of 180 samples were collected and transported to the laboratory on ice the same day they were collected. For the 16S rRNA gene analysis, duplicate samples from each point were combined in 500 mL sterile bottles and thoroughly mixed. Then, a subsample of 40 mL was transferred into a sterile 50-mL tube (BD Falcon, Schaffhausen, Switzerland) and centrifuged for 10 min at 5000× *g* at 4 °C, and the supernatant was discarded, leaving approximately 600 µL of residual liquid. The pellets were frozen at −80 °C until DNA extraction. Water temperature (Temp), pH, dissolved oxygen (DO), and pH were measured on site from each sample using a handheld multiparameter water quality sonde (YSI Inc., Yellow Springs, OH, USA/Xylem Inc., Washington, DC, USA). Other variables, including turbidity, nitrate (NO_3_^−^), sulfate (SO_4_^2−^), phosphorus (P), and ammonia (NH_3_), were determined from another subsample (250 mL) according to the Brazilian standard issued by the Environment National Council (CONAMA resolution 357/2005). Figure 1 provides the data of the sample sites.

### 2.2. DNA Isolation and Library Preparation

Total genomic DNA was extracted from 200 µL resuspended pellets using the PowerSoil DNA kit (MO BIO Laboratories, Carlsbad, CA, USA), according to the manufacturer’s instructions. To minimize potential bias during DNA extraction, each sample was extracted in duplicate and then pooled to quantify their DNA yield with a Qubit fluorometer (Invitrogen, Waltham, MA, USA). Sterile reagent grade type I water was used as a negative control for DNA extraction. The extracted DNA from each sample and the negative control was subjected to PCR amplification of the V3-V4 variable region of the 16S rRNA gene using the previously published primers Bakt_341F/Bakt_805R [33] and conditions previously described by our group [34,35]. A negative control containing all reagents except the template DNA was included with each set of reaction mixtures. Library preparation and massively parallel sequencing (MPS) were performed according to the manufacturer’s protocol for paired-end sequencing using MiSeq Reagent Kit v3, as previously reported [5,34,35].

### 2.3. Detection of Toxin-Producing Cyanobacterial Genes

Three regions of the microcystin synthetase (*mcy*) gene cluster were selected to search for potential microcystin (MC) producers in the Billings samples. Representative samples (*n* = 15) with cyanobacteria of >40% of their bacterial communities were selected for the investigation. A different set of specific published primers designed to detect mcyA (mcyA-Cd_1F; 5′-AAA ATT AAA AGC CGT ATC AAA-′3 and mcyA-Cd_1R; 5′-AAA AGT GTT TTA TTA GCG GCT CAT-′3) [36], mcyD (mcyDF; 5′-GAT CCG ATT GAA TTA GAA AG-′3 and mcyDR; 5′-GTA TTC CCC AAG ATT GCC-′3), and the mcyE gene (mcyE-F2; 5′-GAA ATT TGT GTA GAA GGT GC-′3 and mcyE-R4; 5′-AAT TCT AAA GCC CAA AGA CG-′3) [37] were used. Amplification of these fragments was conducted using a 50–100 ng DNA template, 2 mM MgCl2, 0.1 mM dNTPs, 0.5 μM of each primer, and 2.5 U high-fidelity *Taq* platinum DNA polymerase (Invitrogen, Carlsbad, CA, USA) in a MgSO_4_ reaction buffer. After an initial denaturation of 5 min at 94 °C, 35 cycles of 30 s at 94 °C, 30 s at 55 °C, 60 s at 72 °C, and a final extension at 72 °C for 5 min were performed. Each PCR included a known cyanobacterial DNA positive control and an interspersed no DNA template negative control. The amplified product was electrophoresed through 1% (wt/vol) agarose gels containing 0.5× Tris Borate EDTA, followed by ethidium bromide staining.

### 2.4. Bioinformatics and Statistical Analysis

Base-calling and data quality were initially assessed on an MiSeq instrument using RTA v1.18.54 and MiSeq Reporter v2.6.2.3 software (Illumina Inc., San Diego, CA, USA). The sequences were analyzed by a pipeline of the 16S Microbiome Taxonomic Profiling (MTP) of the EzBioCloud (https://www.ezbiocloud.net/ (accessed on 19 March 2020)) application update 2019.04.09. Briefly, the processing of raw reads started with quality checking and filtering of low quality (<Q25) reads by Trimmomatic version 0.32 [38]. After QC pass, paired-end sequence data were merged together using the fastq_mergepairs command of VSEARCH version 2.13.4 [39] with default parameters. Primers were then trimmed with the alignment algorithm of Myers and Miller [40] at a similarity cut off of 0.8. Nonspecific amplicons that do not encode 16S rRNA were detected by Nhmmer [41] in the HMMER software package version 3.2.1 with hmm profiles. Unique reads were extracted, and redundant reads were clustered with the unique reads by the derep_fulllength command of VSEARCH [39]. The EzBioCloud 16S rRNA database [42] was used for taxonomic assignment using the usearch_global command of VSEARCH [39], followed by more precise pairwise alignment [40]. Chimeric reads were filtered on reads with <97% similarity by reference-based chimeric detection using the UCHIME algorithm [43] and the nonchimeric 16S rRNA database from EzBioCloud. Following chimeric filtering, reads that could not be identified to the species level (with <97% similarity) in the EzBioCloud database were compiled, and the cluster_fast command [39] was used to perform de novo clustering to generate additional OTUs. Finally, OTUs with single reads (singletons) were omitted from further analysis. All analyses of molecular data were performed on both nonrarefied and rarefied data to 15,766 sequences (the size of the smallest library). Our initial analysis revealed that the most predominant bacterioplankton taxa and their abundances in the rarefied data were very similar to those in the nonrarefied data. To avoid discarding data, we decided to run our analysis on nonrarefied data. The ACE [44], Chao1 [45], and jackknife [46] α-diversity indices were used to calculate the bacterial richness, and the Shannon [47], Simpson function [47], and NPShannon [48] indices were used to estimate the bacterial evenness in each group using the Wilcoxon rank-sum test. β-diversity was calculated using the Bray–Curtis dissimilarity distance [49] to reveal differences between surface and bottom layer bacteriomes based on their OTU data. The predicted profiles were categorized into clusters of Kyoto Encyclopedia of Genes and Genomes (KEGG) orthology and pathways. To determine whether the reservoir bacterial compositions are affected by depth status, we performed a principal coordinate analysis (PCoA) at the genus level using the Bray–Curtis dissimilarity distance. A pairwise permutational multivariate analysis of variance (PERMANOVA) test was computed to determine whether there were differences in the bacterial communities between the two layers. The enrichment in the assigned taxonomic and functional profiles of the two groups was defined by linear discriminant analysis (LDA) of the effect size (LEfSe) algorithm. All of the above analyses were carried out in ChunLab’s bioinformatics cloud platform, EzBioCloud 16S-based MTP. Statistically significant differences in physicochemical characteristics between both layers were determined by using Student’s t tests and visualized in GraphPad Prism software V8. The correlations between bacterial community diversity (richness, evenness, and OTUs) and water properties were assessed using bootstrap-based confidence limits (100 iterations) and the Pearson correlation coefficient in a principal component analysis (PCA) implemented in the Past 4.07B package [50]. Uncorrected and corrected *p* values of≤ 0.05 were used for all statistical analyses.

For the detection of bacterial pathogens, we considered any bacteria potentially pathogenic if at least one species with a minimum abundance of 10 strains of any genus was categorized as biosafety level 2 or 3 by the American Biological Safety Association (https://my.absa.org (accessed on 7 May 2020)). The analyses were performed based on uncultivated microorganisms.

## 3. Results

### 3.1. Physicochemical Characteristics of Water Samples

**Figure 2:**
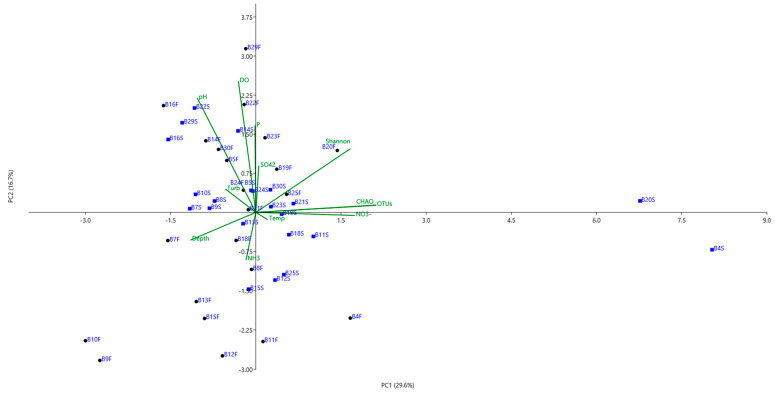
Bootstrap observation biplots derived from the principal component analysis (PCA) of the Billings water physicochemical properties and α bacterial diversities represented by the chao1 and Shannon indices based on 100 resamples of the data. The two principal components (PC1 and PC2) explained 46.3% of the total variation in the environmental data. Temperature (Temp), dissolved oxygen (DO), phosphorus (P), ammonia (NH^3+^), and nitrate (NO^3-^). Green lines represent the correlation coefficient between the PC scores and each environmental parameter. Black dots represent sampling points from the bottom layers, and blue squares represent sampling points from the surface water.

The physicochemical properties of water samples collected from the surface and bottom layers of the Billings Reservoir at 30 different locations are demonstrated in Table 1 and Table 2. The samples were collected in July 2019 when the temperature was above 20 °C and the season was mildly dry. The temperature from both layers ranged from 18.8 °C to 22.1 °C, and DO ranged from 3.5 to 9.5 mg/L. The higher DO levels at some sampling locations are most likely due to wind and aeration. Both temperature and DO parameters were significantly higher (*p* < 0.05) in surface water than in the bottom layers (average temperature: 21.1 °C vs. 20.7 °C, averaged DO: 9.5 mg/L vs. 8.4 mg/L), whereas phosphorus concentrations showed the opposite pattern. The concentrations of ammonia and nitrate along the reservoir showed no obvious difference between the surface and bottom layers. PC1 explained a large proportion of the total variance (29.6%), and the proportion increased to 46.3% when PC2 was added (Figure 2). The variables contributing most to the PC1 were CHAO (r  =  0.50) and NO_3_^−^ (r  =  0.41), while those contributing most to the PC2 were DO (r  =  −0.6) and PH (r  =  −0.52). The bacterial diversity and OTU relative abundance in the Billings Reservoir were positively correlated with the NO^3−^ concentration.

### 3.2. Bacterial Community Structure

**Figure 3:**
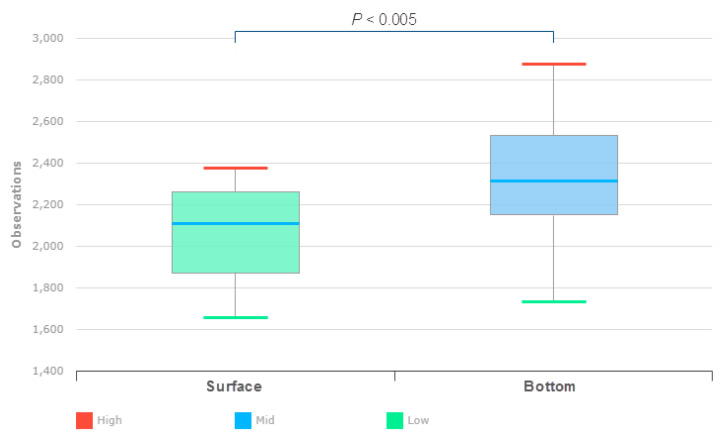
Bacterial community alpha diversity as calculated by the phylogenetic diversity index of water samples from the surface and bottom layers. The error bars indicate the mean with standard error.

Of the 30 locations, 22 paired water samples from the surface and bottom layers were successfully amplified, sequenced, and submitted for 16S rRNA analysis. Up to 100,000 MPS reads from each sample were uploaded, quality controlled, and profiled by the EzBioCloud tool. The trimming-based quality control removed 30,778 and 24,323 low quality amplicons from the surface and bottom layer samples, respectively. The taxonomic approach detected and removed 79,629 and 101,093 nontarget amplicons in the surface and bottom layer samples, respectively. A total of 392,135 and 51,426 chimeric amplicons were identified and removed from the surface and bottom layer samples, respectively. The overall quality assessment and trimming steps resulted in 1,468,033 (Min: 15,766 in B7S; Max: 87,913 in B21S) and 1,861,126 (Min: 35,189 in B22F; Max: 96,434 in B12F) valid reads in surface and bottom layers water, respectively. The distribution of sequence lengths produced agreed with the amplicon length (average 427 bp) of the 16S rRNA. The Good’s coverage estimator of the OTUs in the surface and bottom layer samples ranged from 99.4 to 99.9% and 99 to 99.8%, respectively (Supplementary Tables S1 and S2). Rarefaction curves reached stable values, indicating that the diversity of bacterial populations in both groups was sufficiently covered by the generated sequences (data not shown). The number of OTUs per sample ranged from 1063 to 5919 and from 305 to 2150 in the surface and bottom layer samples, respectively. The α diversity of bacteria in the surface and bottom layers of the reservoir was compared by both richness (i.e., the observed OTUs, ACE, Chao1, and jackknife index) and evenness indices (i.e., the NPShannon, Shannon, Simpson, and phylogenetic diversity index). The results showed that, with the exception of the phylogenetic diversity index, no α indices differed significantly between the bacterial communities from both layers. The phylogenetic diversity index was significantly higher in the bottom layers than in the surface layers (Wilcoxon rank-sum test; *p* < 0.005) (Figure 3). The principal coordinate analysis of the β-diversity d results revealed no significant (Wilcoxon rank-sum test; *p* = 0.131) similarities between the bacterial communities from the reservoir’s surface and bottom layers.

### 3.3. Identification of the Billings Bacteriome in Surface and Bottom Layer Samples

**Figure 4:**
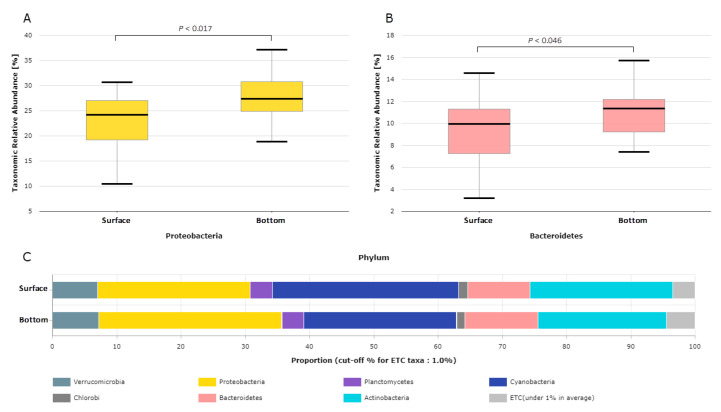
The relative abundance of (**A**) *Proteobacteria* and (**B**) *Bacteroidetes*, and (**C**) relative abundance of bacterial composition at the phylum level of the surface and bottom layers.

**Figure 5:**
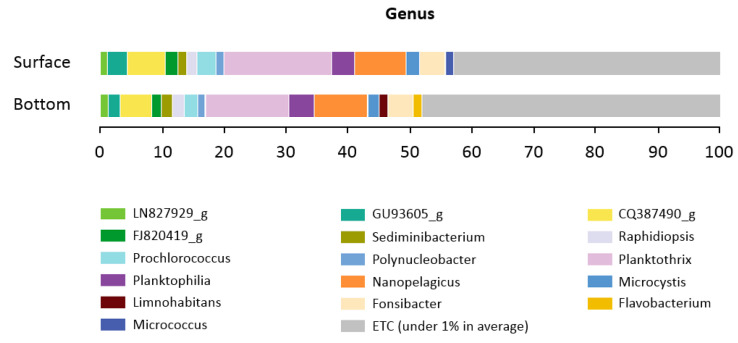
Relative abundance of bacterial composition at the genus level of the surface and bottom layers.

Seven phyla (*Cyanobacteria*, *Proteobacteria*, *Actinobacteria*, *Bacteroidetes*, *Verrucomicrobia*, *Planctomycetes*, and *Chlorobi*) were found to be abundant in samples from both layers. The relative abundance of taxa fluctuated among surface and bottom layer samples at the phylum level, as shown in Supplementary Figures S1 and S2. *Cyanobacteria* predominated the bacteriome of the water in the Billings Reservoir, but the individual composition datasets showed a significant amount of variation. The proportion of *cyanobacteria* ranged from 1.4% (B04S) to 71.1% (B09S) in the surface layer, and from 0.4% (B04F) to 44.6% (B09S) in the bottom layer. The proportion of the phylum *Proteobacteria* ranged from 10.2% (B09S) to 47% (B04S) in the surface layers and from 18.9% (B09S) to 56% (B04S) in the bottom layers. *Proteobacteria* and *Bacteroidetes* were less abundant in the surface layers compared to the bottom layer of the samples (Figure 4). The most common classes in all samples were *Chroobacteria*, *Actinobacteria*, *Betaproteobacteria*, *Alphaproteobacteria*, *Sphingobacteria,* and *Acidimicrobiia* (Supplementary Tables S3 and S4). At the order level, 21.9% and 16.9% of OTUs were assigned to *Oscillatoriales* in the surface and bottom layers, respectively. The order *Planktophila* accounted for >12% of all detected OTUs from Billings *cyanobacterial* sources, followed by the order *Burkholderiales,* which accounted for >24%. At the genus level, 20.2% and 14.5% of OTUs were assigned to the *Planktothrix* genus within the *Microcoleaceae* family in the surface and bottom layers, respectively, and >8% OTUs were assigned to the *Nanopelagicus* genus within the family *Nanopelagicaceae* in both layers (Figure 5). Among the *Cyanobacteria, Chroobacteria* were the most abundant group in the reservoir, with a median relative abundance of 18.96% and 18.08% of all OTUs in the surface and bottom layer samples, respectively. Differentially abundant taxa between the two water samples were identified using the LEfSe algorithm with a minimum LDA score of 2.0 [51]. This analysis revealed 126 taxa, which included 11 classes, 24 families, 29 genera, 24 orders, 6 phyla, and 32 species. Of these, two families, four genera, one order, and nine species were significantly abundant and discriminative between the groups (FDR-adjusted *p* value < 0.02. Supplementary Table S5). The relative abundance of bacteria from the genera *Stenotrophomonas, Achromobacter, Comamonas, Pseudomonas, Acinetobacter*, and *Schlesneria* was significantly higher in the bottom layers than in the surface samples (FDR-adjusted *p*-value < 0.05).

### 3.4. Prediction of Functional Biomarkers

An LEfSe analysis was conducted to predict the most pertinent functional pathways that shape the Billings bacteriome between both layers. The software package PICRUSt2, implemented in the EzBiocloud online tool, was used to infer the content of bacterial genes from the 16S rRNA data and aggregate the relative abundance of functional genes into metabolic pathways. From all OTUs detected in matched samples, 83 KEGG orthology (KO) terms were predicted. Of these, four differentially abundant (FDR-adjusted *p* < 0.05) KO terms between surface and bottom layer water were identified (Supplementary Table S6), and all had LDA effect sizes <3. Most of the differentially abundant predicted KO terms, including protein metabolism, signaling and cellular processes, and cell motility were most abundant in bottom layer samples. The pathway of the flagellar assembly was also enriched in the bottom water layer group, based on the cutoff of uncorrected *p* < 0.02 instead of FDR corrected <0.89, implying that the growth environment for the bacterial communities in the bottom layer water was much higher than that of the surface layer water (LDA = 2.24). PICRUSt module analysis demonstrated that the microbiota in the bottom layers exhibits increased biosynthesis of tetrahydrofolate (M00841) and C21-steroid hormone (M00109), while the surface microbiota showed an increased use of the Mce transport system (M00670).

### 3.5. Search for Predefined Bacterial Groups, Pathogens, and Cyanotoxin Genes

Screening for pre-defined bacterial groups in the surface and bottom layers of the Billings Reservoir revealed important taxa associated with the human gut, including the phylum *Proteobacteria* (surface; median relative abundance value, 24.7%, bottom layer; median relative abundance value, 27.7%). The search for bacterial pathogens in both layers identified several bacterial genera known to be human, animal, and/or plant pathogens, including *Flavobacterium, Legionella, Staphylococcus, Pseudomonas, Aeromonas, Acidovorax*, and *Acinetobacter. Flavobacterium* (surface; median relative abundance value, 0.65%, bottom layer; median relative abundance value, 0.94%) and *Legionella* (surface; median relative abundance value, 0.21%, bottom layers; median relative abundance value, 0.19%) were the predominant genera.

The DNA of the three selected genes involved in the biosynthesis of *mcy* (*mcyA*, *mcyE*, and *mcyD*) was successfully amplified in all analyzed samples, confirming the genetic potential of the strains in the reservoir to produce microcystin (data not shown).

## 4. Discussion

### 4.1. Distribution and Diversities of Bacterial Communities along the Billings Reservoir

The bacterial diversity in surface water from the Billings Reservoir has recently been studied, with an emphasis on bacterial structure; nonetheless, detailed local bacterial heterogeneity remains unknown. In this study, we investigated the distribution of bacterial communities in the surface and bottom layers of the Billings Reservoir, as well as the environmental conditions that influence their composition. Our results demonstrated that the overall α bacterial diversity represented by the Shannon index was relatively higher in the Billings Reservoir than in other studied freshwater systems: Itupararanga Reservoir, State of São Paulo, Brazil [52], in Pavin Lake [53], and Mayinghai, Pipahai, and Gonghai Lakes in China [54]. Perhaps the greatest value of biodiversity is attributed to intensive anthropogenic disturbance in the reservoir, which may have resulted in increased nutrient discharges, resulting in greater nitrogen and phosphorus concentrations, as indicated in this study. Dias et al. [55] demonstrated that the influence of surrounding human activities, as measured by the human footprint index, had a significant impact on the functional diversity and trait composition of fish assemblages in the Billings Reservoir. It is possible that other variables not included in our study could have contributed to the variability of the bacterial community in the Billings Reservoir.

The observed bacterial community structure and diversity indices in this study were comparable to those in other reservoirs [56]. The taxonomical composition revealed that the *Cyanobacteria* phylum was the most abundant in the community, followed by *Proteobacteria* and *Actinobacteria*. These results are consistent with other Brazilian studies that describe bacterial communities in the Amazon basin [57,58,59] and Tocantins River [60], but are somewhat different from those previously described for reservoirs in China. For instance, Qu J et al. [56] used the 16S rDNA Illumina approach to show that *Firmicutes*, *Proteobacteria*, *Cyanobacteria*, *and Bacteroidetes* were the dominant bacterial phyla in the Miyun Reservoir, which is considered the largest man-made reservoir in North China. These differences in dominating phyla may be due to a variety of environmental factors, such as air, soil, and water pollution, rainfall-induced nutrient fluctuations, and changes in local conditions. The difference may also be attributed to the use of distinct DNA extraction methods and/or primer selection [61] or different 16S rDNA gene bioinformatic pipelines. Although the bacterial richness indices varied little between the surface and bottom layer samples in this study, some specific bacterial groups showed a clear difference. For instance, members of the *β-Proteobacteria* and *γ-proteobacteria*, *Acidobacteria*, *Bacteroidetes*, and *Sphingobacteriales* were significantly more abundant in the bottom layer than in the surface water. Because the water flow within certain layers could contribute significantly to the displacement of bacterial populations, it is reasonable to assume that the latter could constitute a contribution to the observed bacterial community differences between the layers. It is also conceivable that the phosphorus availability in the bottom layers compared to the surface water could positively influence the growth rates of these bacterial groups [62].

In addition to the seven dominant phyla in the Billings Reservoir, there were approximately 3.5% and 4.4% of unclassified taxa in the surface and bottom layers water, respectively, indicating that as-yet-unidentified bacterial populations with unknown metabolic functions are an important part of the reservoir bacteriome. These findings warrant further metagenomics analysis.

### 4.2. Dominant Taxonomic Groups in the Bacterioplankton Community of the Reservoir

Members of *Cyanobacteria* were detected as the most dominant genus, and all *Cyanobacteria* blooms tested here were toxic. These results lend further support to previous studies that found cyanobacterial communities in Brazilian semiarid reservoirs [32,63,64,65,66,67]. The presence of these bacteria in high relative abundance in the reservoir can be linked to uploading nutrients such as ammonia and an increase in water temperature (20.7 °C) at the time of sampling. The OTUs of these bacteria have been shown to outcompete other planktonic microbes for nutrients in eutrophic systems [68]. A previous study on the Neuse River, North Carolina, conducted by Paerl, recommended that a reduction of 30–40% of NO_3_ had the optimum power of minimizing *M. aeruginosa* as a dominant phytoplankter [69]. Concerning temperature, there is a consensus among researchers that water temperatures below 20 °C are generally considered unfavorable for the development of common water bloom-forming genera such as *Dolichospermum* and *Microcystis*. In contrast to this, our findings showed widespread *Microcystis* and *Planktothrix* in the reservoir. The presence of more *Cyanobacteria* in freshwater systems has a variety of impacts, including oxygen depletion, fish mortality, and toxicity, which offer a variety of health risks [70]. Because the *Cyanobacteria* detected in this study are capable of producing toxins, their presence in the reservoir poses a huge threat to animal and human health.

In this study, *Proteobacteria* constituted a sizable fraction of the bacterial community in the reservoir, which is consistent with previous reports [71,72]. Despite the dominance of *Proteobacteria* in both layers, a significant difference in the composition of these members was observed, as detailed in the taxonomic analysis. *Betaproteobacteria* were the most frequently detected *Proteobacteria* group in this work, which agrees with other studies [73,74]. Microorganisms belonging to the *Betaproteobacteria* class have been associated with anthropogenic activities [75]. The alpha and *Gammaproteobacteria* found in both layers probably indicate an increase in organic and inorganic inputs, as well as phytoplankton production [62,73].

The *Actinobacteria* phylum was the reservoir’s third most abundant OTU phylum. Members of this phylum are among the most abundant groups in freshwater habitats [73,76]. The relative abundance of these microbes is inversely correlated to that of *Cyanobacteria*, which cause prolonged and irretrievable ecological disturbances to freshwater ecosystems and serve as sentinels of impending ecological damage [77,78]. The OTUs of *Actinobacteria* were less abundant than those of *Proteobacteria*, which comprised 23.7% and 28.5% of the total bacterial relative abundance in the surface and bottom layer water of the Billings Reservoir, respectively. *Actinobacteria*, which are well-recognized soil bacteria [79], are frequently detected in oligotrophic freshwater habitats [80] and are often associated with oligotrophic ecosystems [81]. They have long been known to produce pigments that protect them from UV radiation, which may easily penetrate deep into a freshwater habitat [73].

### 4.3. Bacterioplankton Community Diversity between the Surface and Bottom Layers

The alpha diversity analysis conducted between the surface and bottom layers revealed high phylogenetic diversity indices of the bacteriome inhabiting the bottom layers. This result may indicate that bacterial communities in the bottom layers have experienced high diversification rates or the successful immigration of multiple lineages. One factor that may promote higher bacterial diversification in the bottom layers is that this habitat is possibly less extreme than that of the surface layers and permits easier radiation [82]. An examination of Billings water did not reveal significant differences in bacterial community β-diversity between the two layers, suggesting that a core bacteriome exists between both layers of the reservoir.

### 4.4. Potential Functions and Pathways of Bacterial Communities in the Reservoir

Application of the LEfSe method demonstrated the presence of *Stenotrophomonas species* as the most significant specific biomarkers in the bottom layers. These species play an important ecological role in the nitrogen and sulfur cycles, and several *Stenotrophomonas species* can engage in beneficial interactions with plants, promoting growth and protecting plants from attack [83]. Because the reservoir contains a large number of *Cyanobacteria*, the death of many of these bacteria could result in the release of a high contents of sulfur-containing amino acids from their cells [84], resulting in sulfur-rich water and thus explaining the detection of *Stenotrophomonas species*. However, these conclusions must be interpreted with caution given the serious limitations of deducing the biofunctions of the reservoir bacteria from 16S rRNA gene sequencing and the potential risk of bias.

In addition to taxonomic compositions, we identified a pathway containing genes encoding for flagellar assembly that were abundant in the Billings 16S metagenome. The occurrence of flagellar assembly genes within the bottom layers indicates that the reservoir contains bacterial communities that utilize flagella for locomotion. It is possible that the adhesion process of these bacteria was influenced by environmental factors such as pH and higher levels of metals introduced into the reservoir because of the rapid expansion of industry and intense domestic activities.

### 4.5. Occurrence of Pathogenic Bacteria

Many developing countries are suffering widespread contamination of their freshwater supplies by bacterial pathogens, which have been linked to epidemics of numerous waterborne diseases [85]. In the current study, we found that pathogenic bacteria were ubiquitous across all the sampled waters in the Billings Reservoir. Among these pathogens, the *Flavobacterium* and *Legionella* genera were the most prevalent in both layers. Although most *Flavobacteria* are harmless, some are opportunistic or true pathogens that cause diseases in animals, plants, and humans [86,87,88]. The majority of *Legionella species* are considered pathogenic and have been reported as one of the leading bacterial etiological pathogens of waterborne outbreaks in the United States between 2007 and 2009 [89].

## 5. Limitations and Conclusions

The main limitation of this study is that we restricted the analysis to samples at a single point in time because water samples from the bottom layers were collected only once. While the spatiotemporal data from surface water are still incomplete, the analysis of the data that is now available is well underway. In addition, we used bacterial DNA genomics for this investigation, which would have revealed the presence of bacterial populations regardless of whether they were dead or alive, culturable cells, or nonculturable cells. Another limitation, inherent in 16S rRNA diversity surveillance by the Illumina MiSeq platform, is the fact that the taxonomic and ecological resolution of the 16S rRNA gene V3-V4 region is insufficient for accurately identifying pathogenic bacteria and strains with flagella genes to species levels, as revealed in functional analysis. These results should be amenable to robust metagenomic data, RNA sequencing, and cultivable fractions to expand our understanding of the bacterial strains associated with the reservoir, their genes, and metabolic pathways. That being said, our findings do reveal the extent of bacterial diversity in the reservoir, the health hazards for household drinking water, and the effects of the direct use of water by residents of the reservoir communities.

To summarize, the toxin-producing *Cyanobacteria* phylum was found to be the most abundant in the community, followed by Proteobacteria and Actinobacteria. Ammonia and high water temperature are two potential contributors to the abundance of *Cyanobacteria* in the reservoir. According to functional prediction analyses, genes associated with cell motility, such as flagellar assembly, were abundant in the Billings bacteriome.

In conclusion, this study provides important information about the numerous bacteria inhabiting the Billings Reservoir and the combination of environmental factors that shape their structure. These results may help pave the way for future studies devoted to controlling and improving the water quality in the Billings Reservoir, which is facing rapid urban development and urbanization.

## Figures and Tables

**Table 1:** Physicochemical characteristics of water samples from the surface of the Billings Reservoir.

Sample ID	DO * mg/L	Temp	pH	Average Water Depth (km)	Turbidity (NTU)	Sulfate mg/L	Phosphorus mg/L	Ammonia mg/L	Nitrate mg/L
B1S	4	20.5	7.3	9.5	3.1	0.4	0.1	2.4	0.9
B2S	7.1	21.2	8.3	5.5	3.1	0.5	0.2	2.8	1.1
B3S	5.7	21.2	7.5	3.2	2.5	0.4	0.1	2.8	2.2
B4S	5.5	22.1	7.0	2.1	1.9	0.6	0.0	2.7	7.4
B5S	7.4	21.5	8.1	6.9	2.5	0.4	0.2	2.7	0.9
B6S	8.3	21.6	8.7	12.8	4.3	0.3	0.3	2.2	1.2
B7S	7.5	21.2	8.2	12.4	6.9	0.3	0.1	2.6	0.9
B8S	7.3	21.1	7.9	10.1	15.0	0.4	0.3	2.5	1.2
B9S	7.2	21.2	7.6	9	11.5	0.7	0.3	2.5	1
B10S	7.9	21.5	7.9	7.6	18.5	0.3	0.8	2.9	2.2
B11S	5.8	21.5	7.6	5.9	12.0	0.6	0.1	3	1.8
B12S	4.3	21.2	7.5	8.1	14.1	0.5	0.0	2.6	1.4
B13S	6.7	20.8	7.8	10.4	10.0	0.5	0.1	2.7	1.9
B14S	9.5	20.9	8.5	3.9	12.0	0.54	0.1	3.3	1.8
B15S	4.7	20.9	7.5	13.6	6.2	0.56	0.0	2.7	1.7
B16S	9.2	21	8.6	10.1	12.9	0.4	0.1	2.8	1.3
B17S	7.9	20.6	7.7	6.9	11.8	0.6	0.1	2.7	2.3
B18S	6.5	20.5	7.5	8.9	18.0	0.4	0.2	2.7	2.65
B19S	6.3	20.5	7.6	6.1	7.8	0.5	0.1	2.1	1.3
B20S	6.5	20.9	7.3	4.5	10.5	0.3	0.1	2.6	2.9
B21S	7.7	20.9	7.6	7	12.0	0.5	0.2	2.8	2.2
B22S	9.4	20.6	8.3	5.4	11.0	0.5	0.2	2.1	1.1
B23S	9	21	7.3	5.8	6.5	0.4	0.2	2.4	1.8
B24S	7.9	21.3	8.0	5.5	8.1	0.5	0.0	2.9	1.02
B25S	5.8	21.8	7.6	6.9	5.1	0.5	0.1	3.4	1.98
B26S	7.6	21.6	8.0	6.8	6.5	0.4	0.1	3.2	0.9
B27S	9.2	20.8	7.4	3.2	5.3	0.3	0.1	2.4	0.8
B28S	9.3	20.6	7.9	7.1	4.8	0.4	0.1	2.8	0.9
B29S	9.3	21.6	8.9	8.7	5.3	0.5	0.1	2.9	1.2
B30S	8.1	20.9	7.8	10.2	8.3	0.4	0.2	2.3	2.8

* Dissolved oxygen.

**Table 2:** Physiochemical characteristics of water samples from the bottom layer of Billings reservoir.

Sample ID	DO * mg/L	Temp	pH	Average Water Depth (km)	Turbidity (NTU)	Sulfate mg/L	Phosphorus mg/L	Ammonia mg/L	Nitrate mg/L
B1F	3.7	20.6	7.4	9.5	4.1	0.4	0.1	2.5	1.1
B2F	6.4	20.8	8.3	5.5	3.0	0.5	0.2	2.3	1.2
B3F	5.1	19.8	7.2	3.2	3.9	0.4	0.2	2.8	1.5
B4F	4	18.8	7	2.1	1.2	0.1	0.1	2.4	1.2
B5F	7.1	21.1	8.2	6.9	3.0	0.6	0.1	2.3	0.05
B6F	7.1	21.7	8.3	12.8	4.4	0.4	0.1	3.2	0.09
B7F	5.6	21.2	8.1	12.4	6.3	0.5	0.1	2.8	1.2
B8F	4.6	21.1	7.9	10.1	8.1	0.2	0.1	2.2	1.1
B9F	4.5	20.9	7.2	9	9.8	0.4	0.0	2.4	0.9
B10F	4.8	21.2	7.6	7.6	13.6	0.5	0.1	3.7	0.8
B11F	3.5	21.2	7.6	5.9	10.3	0.2	0.0	3.1	1.1
B12F	3.5	20.9	7.4	8.1	6.7	0.3	0.0	2.8	0.8
B13F	4.9	20.8	7.7	10.4	10.3	0.5	0.0	3.1	1.2
B14F	8.3	21	8.3	3.9	31.9	0.3	0.0	2.5	0.9
B15F	4.3	20.8	7.5	13.6	6.1	0.5	0.1	2.7	1.2
B16F	8.4	20.6	8.5	10.1	11.5	0.4	1.2	2.3	0.09
B17F	6.3	20.5	7.8	6.9	16.5	0.4	1.1	2.6	1.1
B18F	6	20.3	7.6	8.9	13.1	0.4	0.1	2.7	0.09
B19F	5.9	20.5	7.6	6.1	9.9	0.8	1.1	2.6	1
B20F	5.5	20.4	7.5	4.5	9.9	0.6	2.1	2.4	2.7
B21F	6.6	20.5	7.7	7	8.4	0.3	0.9	2.8	1.1
B22F	7.8	20.5	8.4	5.4	10.2	0.6	1.2	2.7	1.6
B23F	6.9	20.9	8.1	5.8	8.2	0.5	1.3	2.3	1.8
B24F	7.1	20.7	8	5.5	6.8	0.4	0.9	3	2.1
B25F	6.6	20.9	7.9	6.9	6.2	0.5	1.2	3.5	1.9
B26F	7.6	21.3	8	6.8	7.8	0.5	1.9	2.9	2.3
B27F	8.3	20.5	7.8	3.2	3.4	0.6	2.1	3.1	1.9
B28F	7.6	20.5	7.9	7.1	7.2	0.4	1.9	2.9	1.6
B29F	8.2	20.6	8.6	8.7	7.7	0.5	2.1	2.4	1.1
B30F	7.7	20.5	8	10.2	7.6	0.5	0.9	2.3	1.3

* Dissolved oxygen.

## Data Availability

All sequence data described here are available in the online Zenodo repository: https://doi.org/10.5281/zenodo.4751698 (accessed on 12 May 2021).

## Supplementary Materials

The following supporting information can be downloaded at: https://www.mdpi.com/article/10.3390/life12081280/s1, Figure S1: The relative abundance of dominant taxa at the phylum level in surface water samples. Figure S2: The relative abundance of dominant taxa at the phylum level in bottom-layer samples. Table S1: Valid reads sequenced for each matched sample from the surface layer and alpha diversity indices at the 97% OTU level of 16S rRNA gene fragments. Table S2: Valid reads sequenced for each matched sample from the bottom layer and alpha diversity indices at the 97% OTU level of 16S rRNA gene fragments. Table S3: Count of the individual surface layer bacterial microbiota of Billings reservoir on a phylum, class, order, family, and genus levels. Table S4: Count of the individual bottom layer bacterial microbiota of Billings reservoir on a phylum, class, order, family, and genus levels. Table S5: Differentially abundant taxa between surface and bottom layer water samples identified using LEfSe analysis (minimum LDA score: 2.0). Table S6: Functional biomarkers computed by the LEfSe analysis.
