# Supplementary figures and images for: Characterization of Bacterial Communities from the Surface and Adjacent Bottom Layers of Water in the Billings Reservoir
Source: Life (Basel). 2022 Aug 22;12(8):1280. doi: 10.3390/life12081280 (PMC9409723; doi:10.3390/life12081280)

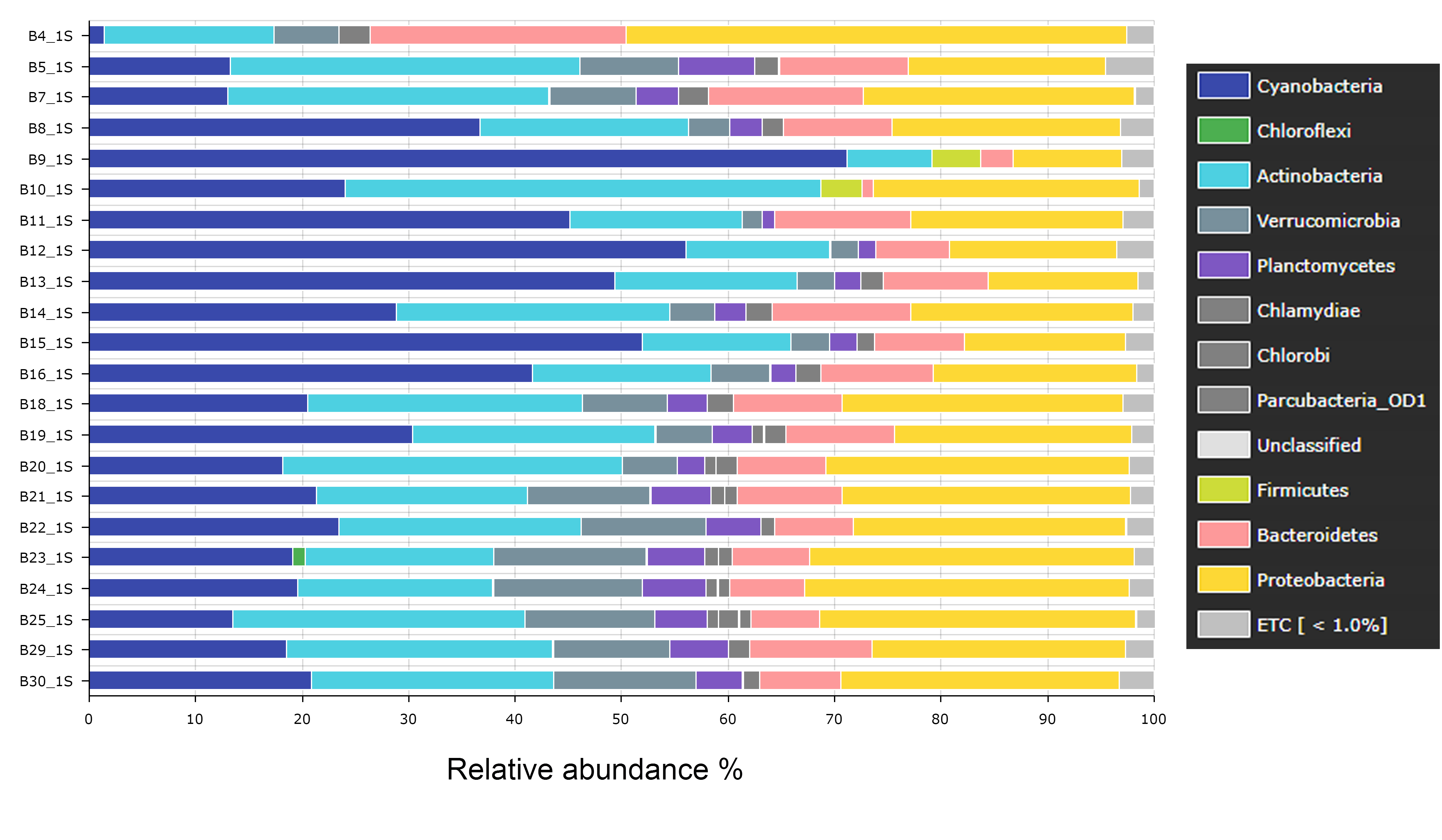

Supplement: Supplementary file 1 [file life-12-01280-s001.zip › Supplementary figures/supplementary figure S1.tif]

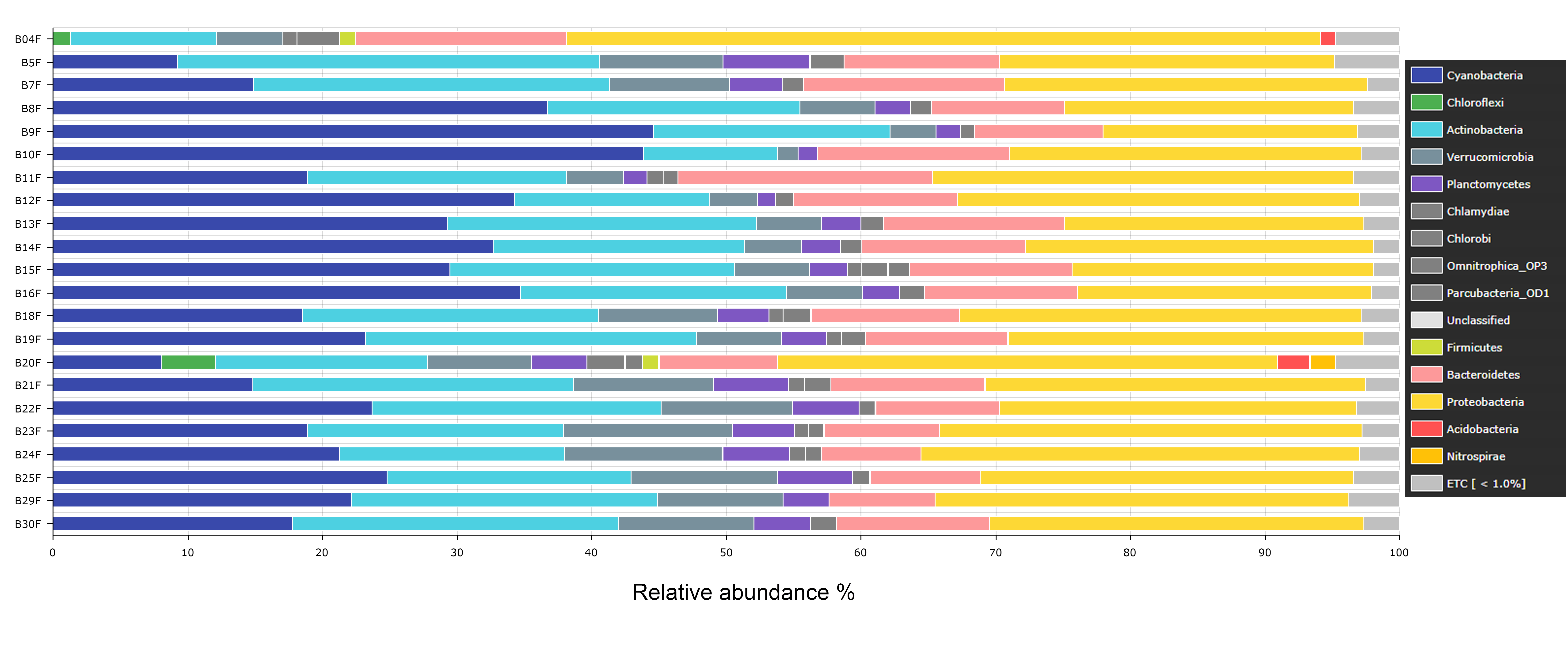

Supplement: Supplementary file 1 [file life-12-01280-s001.zip › Supplementary figures/supplementary figure S2.tif]
